# Phase II study of dose‐adjusted gemcitabine, dexamethasone, cisplatin, and rituximab in elderly relapsed diffuse large B‐cell lymphoma patients

**DOI:** 10.1002/jha2.111

**Published:** 2020-10-15

**Authors:** Satoshi Yamasaki, Akiko Kada, Ilseung Choi, Hiroatsu Iida, Naohiro Sekiguchi, Naoko Harada, Morio Sawamura, Takeshi Shimomura, Takuya Komeno, Takahiro Yano, Isao Yoshida, Shinichiro Yoshida, Kazutaka Sunami, Terutoshi Hishita, Hiroshi Takatsuki, Koichi Ohshima, Morishige Takeshita, Akiko M. Saito, Hiromi Iwasaki, Hirokazu Nagai

**Affiliations:** ^1^ Department of Hematology and Clinical Research Institute National Hospital Organization Kyushu Medical Center Fukuoka Japan; ^2^ Clinical Research Center National Hospital Organization Nagoya Medical Center Nagoya Japan; ^3^ Department of Hematology National Hospital Organization Kyushu Cancer Center Fukuoka Japan; ^4^ Department of Hematology National Hospital Organization Nagoya Medical Center Nagoya Japan; ^5^ Department of Hematology National Hospital Organization Disaster Medical Center Tachikawa Japan; ^6^ Department of Hematology National Hospital Organization Kumamoto Medical Center Kumamoto Japan; ^7^ Department of Hematology National Hospital Organization Shibukawa Medical Center Shibukawa Japan; ^8^ Department of Hematology National Hospital Organization Hiroshimanishi Medical Center Otake Japan; ^9^ Department of Hematology National Hospital Organization Mito Medical Center Ibaraki Japan; ^10^ Department of Hematology National Hospital Organization Tokyo Medical Center Tokyo Japan; ^11^ Department of Hematologic Oncology National Hospital Organization Shikoku Cancer Center Matsuyama Japan; ^12^ Department of Hematology National Hospital Organization Nagasaki Medical Center Omura Japan; ^13^ Department of Hematology National Hospital Organization Okayama Medical Center Okayama Japan; ^14^ Department of Hematology National Hospital Organization Himeji Medical Center Himeji Japan; ^15^ Department of Hematology National Hospital Organization Kokura Medical Center Kitakyushu Japan; ^16^ Department of Pathology School of Medicine Kurume University Kurume Japan; ^17^ Department of Pathology Faculty of Medicine Fukuoka University Fukuoka Japan

**Keywords:** diffuse large B‐cell lymphoma, elderly patients, gemcitabine, quality of life, relapsed, rituximab

## Abstract

High‐dose chemotherapy and autologous stem cell transplantation (ASCT) are too toxic for elderly patients with relapsed or refractory diffuse large B‐cell lymphoma (DLBCL). Therefore, effective and tolerable regimens for elderly patients are urgently needed. The present phase II study assessed the efficacy and safety of dose‐adjusted therapy with gemcitabine, dexamethasone, cisplatin, and rituximab (GDP‐R) in this population. ASCT‐ineligible elderly patients with relapsed or refractory DLBCL received dose‐adjusted GDP‐R in each 28‐day cycle for up to six cycles. The primary endpoint was overall response rate (ORR), and secondary endpoints were complete response (CR) rate, progression‐free survival (PFS), and safety. Thirty‐three patients were enrolled and received dose‐adjusted GDP‐R. The median age was 75 years (range: 68‐87 years). The ORR was 82.8% (90% confidence interval [CI], 67.1‐93.0%), with a CR rate of 58.6% (90% CI, 41.7‐74.1%). At a median follow‐up of 20.9 months, the 2‐year PFS rate was 46.8% (90% CI, 30.7‐61.5%) and the 2‐year overall survival rate was 63.2% (90% CI, 45.8‐76.3%). The most frequently observed grade 4 adverse events were neutropenia (63.6%), thrombocytopenia (57.6%), and lymphocytopenia (39.4%). Dose‐adjusted GDP‐R is a promising salvage regimen for ASCT‐ineligible elderly patients with relapsed DLBCL after rituximab‐containing chemotherapy and warrants further investigation.

## 1 INTRODUCTION

Diffuse large B‐cell lymphoma (DLBCL) is the most common lymphoma subtype (45.3%) in Japan [1], and around 40% of cases occur in patients older than 70 years [2]. High‐dose chemotherapy and autologous stem cell transplantation (ASCT) are standard treatments for younger patients with relapsed or refractory (R/R) DLBCL, but these regimens may be too toxic for elderly patients [3]. Although a few studies have focused on outcomes of relapsed elderly patients, attempts at conventional salvage regimens in older patients often do not result in disease control and have substantial morbidity [4]. Therefore, efficient and tolerable salvage treatment for elderly patients is urgently needed.

Gemcitabine is an analog of cytarabine with more efficient cellular kinetics, including intracellular incorporation, phosphorylation, and retention [5]. Hayashi et al reported that gemcitabine treatment synergistically increased rituximab‐mediated complement‐induced cell activity in vitro and suggested that a combination of gemcitabine and rituximab might enhance the antitumor effects of rituximab against DLBCL because of CD20 upregulation on lymphoma cells [6]. Phase II trials conducted in ASCT‐eligible or ASCT‐ineligible patients suggested that treatment with gemcitabine, dexamethasone, and cisplatin (GDP) could be administered efficaciously and safely, and would be well tolerated [7]. Although GDP has been proposed for ASCT‐ineligible elderly patients with R/R DLBCL, results have not been satisfactory [8, 9]. Therefore, we evaluated the efficacy and safety of the combination of GDP and rituximab (GDP‐R) in ASCT‐ineligible elderly DLBCL patients treated previously with rituximab in a single‐arm phase II trial.

## 2 PATIENTS AND METHODS

### 2.1 Study oversight

This study was a multicenter, open‐label, single‐arm phase II trial (UMIN000015492). It was conducted in 20 Japanese centers belonging to the Japanese National Hospital Organization (J‐NHO). Protocol details have been published previously [10] (Supporting Information). We designed this trial to evaluate dose‐adjusted GDP‐R as salvage chemotherapy for ASCT‐ineligible elderly DLBCL patients previously treated with rituximab. The dose‐adjustment paradigm was designed to reduce age‐related or nonhematological toxicities, which were published previously (Supporting Information). The trial was approved by the ethics boards of all participating centers, and written informed consent was provided by all participants. An independent data and safety monitoring committee monitored the trial every 6 months. The J‐NHO conducting this trial had its own financial support.

### 2.2 Pathology review process

A pathology review was conducted centrally by an expert hematopathologist, who classified all patients according to the World Health Organization classification [11]. A lymphoma phenotype was determined using available tissue blocks and evaluating a standard phenotype panel and, if indicated, cytogenetic and molecular studies, including MYC rearrangements in addition to BCL2 and/or BCL6 rearrangements (detected using fluorescence in situ hybridization [FISH] or standard cytogenetics; Supporting Information), were performed to detect double‐hit lymphomas [12] and confirm DLBCL subtypes using Hans's criteria (germinal center B‐cell‐like [GCB] and non‐GCB subtypes) [13]. Local and regional phenotype data, as well as any cytogenetic or molecular results, were tabulated for review. Then, central and regional expert pathologists rendered the diagnosis and quantitated various pathologic parameters.

### 2.3 Treatment protocol

Eligible patients were aged 65 years or older, with R/R DLBCL, who had received at least three cycles of one standard chemotherapeutic regimen, rituximab, cyclophosphamide, doxorubicin, vincristine, and prednisolone (R‐CHOP). Baseline assessments included physical examination, standard laboratory tests, computed tomography (CT) scanning of the chest, abdomen, and pelvis, and if indicated, a bone marrow biopsy. Eligible patients were required to have measurable disease by CT scan or physical examination, an Eastern Cooperative Oncology Group performance status (PS) of 0‐3, and acceptable hematologic and biochemical parameters. Patients were excluded if they had previously received treatment with gemcitabine and cisplatin, had CNS involvement with DLBCL, had a history of hepatitis B virus, hepatitis C virus, or human immunodeficiency virus infection, or a medical condition that would interfere with the safe administration of the protocol chemotherapy.

Three cycles of protocol therapy were administered every 28 days for up to six treatment cycles, depending on the response and toxicity, and consisted of intravenous gemcitabine 1000 mg/m^2^ of body surface area per day on days 1 and 8 and cisplatin 75 mg/m^2^ on day 1, oral dexamethasone 40 mg per day on days 1 through 4, and rituximab 375 mg/m^2^ intravenously on days 1 through 8, with the expectation that there would be a synergistic effect between gemcitabine and rituximab.

Patients who had not achieved a complete response (CR) or a partial response (PR) after three treatment cycles were permitted to be withdrawn from the study. Treatment was designed to be delivered to patients in an outpatient setting and included a recommended minimum hydration schedule for cisplatin. Each participating center was responsible for determining policies for supportive care after treating patients with dose‐adjusted GDP‐R. Granulocyte colony‐stimulating factor was given daily if neutrophil counts decreased to less than 1000/μL.

### 2.4 Efficacy evaluation

Disease status assessments included physical examinations, CT scans, and bone marrow analysis. Tumor response evaluation was performed according to the Revised Response Criteria for Malignant Lymphoma [14]. CT scans of the neck, thorax, abdomen, and pelvis or positron emission tomography (PET)‐CT was performed every 3 months for up to 24 months or until initiation of alternative DLBCL treatment, whichever came first. The primary endpoint of response rate after three cycles of dose‐adjusted GDP‐R (interim response) was calculated by considering the number of patients achieving a CR or PR, evaluated using PET‐CT among those eligible patients who received at least one cycle of protocol therapy as described previously [10] (Supporting Information). Progression‐free survival (PFS) was defined as the time from the start of treatment to the date of progression. Overall survival (OS) was defined as the time from the start of treatment to the date of death. Adverse events (AEs) from the start of treatment until 6 months after the last treatment were graded according to National Cancer Institute Common Toxicity Criteria version 4.0. Quality of life (QOL) was measured using the QOL Questionnaire for Cancer Patients Treated with Anticancer Drugs (QOL‐ACD) [15] and the SF‐36 health survey [16], and patients were assessed at baseline, middle (M), end of the protocol (E), and 6 months after the end of the protocol (S). A statistically significant change in a QOL score when compared with the baseline score was considered a clinically meaningful change.

### 2.5 Statistical analysis

The sample size calculation was reported previously [10]. Briefly, the number of patients was calculated as 38 based on a binomial cumulative distribution function under the conditions of expected and threshold response rates of 55% and 35%, a one‐tailed significance level of 0.05, and a statistical power of 0.80. With 10% of ineligible patients after registration, the target number of patients was established as 42.

The efficacy analysis set consisted of eligible patients receiving protocol treatment after registration. The safety analysis set was defined as all patients receiving protocol treatment after registration. The point estimation and 90% confidence interval (CI) of the response rate were calculated. The incidence of AEs was calculated. PFS, OS, and time to response were calculated using the Kaplan‐Meier method. The Cox proportional hazards model was used in univariate analysis of PFS‐ and OS‐related factors. OS, according to the interim response after three cycles of treatment (CR + PR vs stable disease [SD] + progressive disease [PD]), was evaluated using the log‐rank test. Changes from baseline in the QOL‐ACD and SF‐36 health survey were determined by the Wilcoxon signed‐rank test. The significance level was .05 in the one‐tailed test for the primary endpoint and .05 in two‐tailed tests. Statistical analyses were performed using SAS version 9.4 (SAS Institute Inc., Cary, NC, USA).

## 3 RESULTS

### 3.1 Patients

Between January 2015 and December 2017, 33 patients older than 65 years with R/R transplant‐ineligible DLBCL were enrolled from 14 hospitals that belonged to the J‐NHO. This study was terminated on December 2017 because of planned enrollment periods. All diagnoses of biopsies were reclassified and DLBCL was confirmed by an experienced hematopathologist. One patient with a transformed follicular lymphoma and one patient without a confirmed diagnosis of a R/R DLBCL were excluded from the efficacy analysis. No patients received ASCT because of the physician's decision based on their advanced age. The median age was 75 years (range: 68‐87 years), and 19 out of 31 (61.3%) patients were male (Table 1). Most patients had stage III or IV disease, a high‐intermediate or high‐risk international prognostic index (IPI), and had either not achieved remission with initial therapy or had recurrence of lymphoma within or after more than 1 year of completing treatment. By Hans's criteria, 25 of 31 patients diagnosed with GCB DLBCL and six with non‐GCB DLBCL were assessed. Regarding pathological markers, 14.2% and 3.5% of patients had BCL‐2‐FISH and c‐Myc‐FISH > 10%, respectively, and one patient was double positive for Bcl‐2‐FISH and c‐Myc‐FISH.

**TABLE 1:** Characteristics of patients with refractory or relapsed diffuse large B‐cell lymphoma

Characteristics	n = 31
Median age (range), years	75 (68‐87)
Sex, n (%) male	19 (61.3)
female	12 (38.7)
ECOG PS, n (%) 0	13 (41.9)
1	15 (48.4)
2	3 (9.7)
Ann Arbor stage at diagnosis, n (%) I/IE	3 (9.7)/2 (6.5)
II/IIE	2 (6.5)/2 (6.5)
III/IIIE	7 (22.6)/1 (3.2)
IV	14 (45.2)
IPI at diagnosis, n (%) low	3 (9.7)
Low‐intermediate	9 (29.0)
High‐intermediate	12 (38.7)
High	7 (22.6)
Immunophenotypical features	
GCB/non‐GCB^a^, n (%)	25 (80.6)/6 (19.4)
P53 (IHC ≥ 20%) status, n (%)	6 of 28 (21.4)
Number of regimens, n (%) 1	25 (80.6)
2	2 (6.3)
≥3	4 (12.5)
Disease status, n (%) PIF	4 (12.9)
Relapse < 1 year after initial therapy	9 (29.0)
≥1 year after initial therapy	18 (58.1)

^a^ Cell of origin subtype using Hans`s criteria.

Abbreviations: CD, cluster of differentiation; ECOG, Eastern Cooperative Oncology Group; FISH; fluorescence in situ hybridization; GCB, germinal center B‐cell‐like; IHC, immunohistochemistry; IPI, international prognostic index; PIF, primary induction failure; PS, performance status.

### 3.2 Treatment outcomes

**FIGURE 1:**
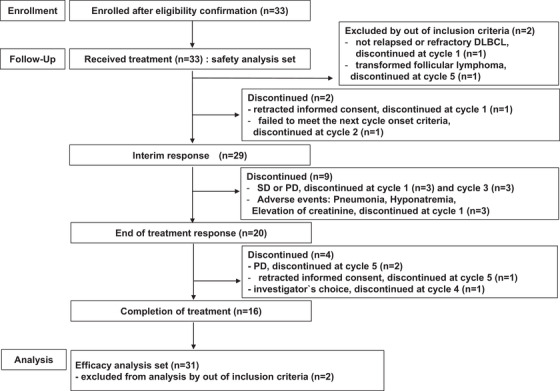
Flow diagram of patients. DLBCL, diffuse large B‐cell lymphoma; PD, progressive disease; SD, stable disease

**FIGURE 2:**
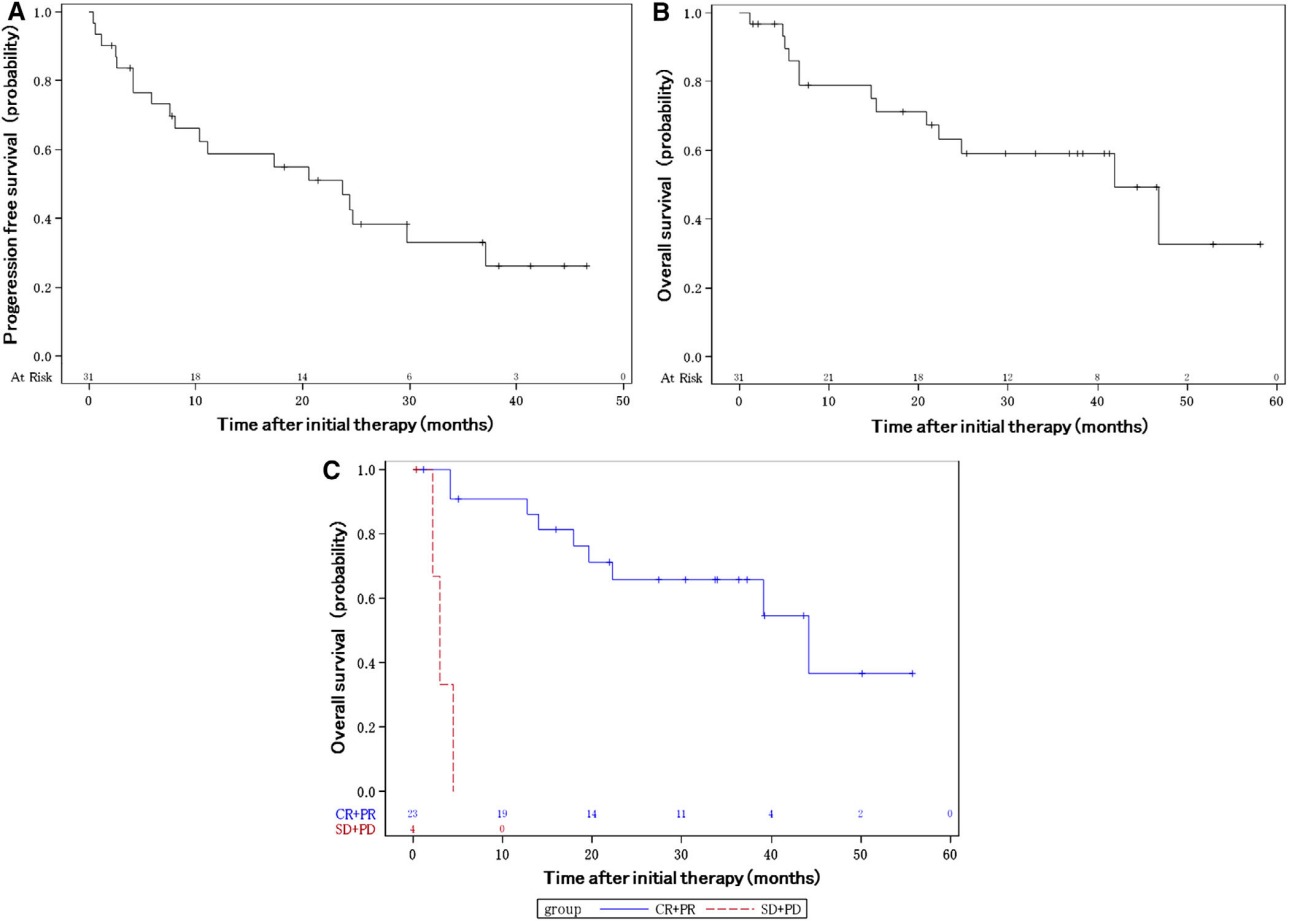
Progression‐free survival and overall survival. Progression‐free survival (PFS; **A**) and overall survival (OS; **B**) in 31 evaluable patients were plotted using the Kaplan‐Meier method. The 2‐year PFS rate and OS rate were 46.8% and 63.2%, respectively (90% confidence interval, 30.7‐65.1% and 45.8‐76.3%). **C,** OS associated with response to treatment (CR + PR vs SD + PD) in 31 evaluable patients was plotted using the Kaplan‐Meier method. There was a significant difference in OS between CR + PR and SD + PD (*P* < .001). CR, complete response; PD, progressive disease; PR, partial response; SD, stable disease

At least one cycle of protocol therapy was administered to 33 patients. The median cycle of GDP‐R was 4 (range: 1‐6). The overall response rate (ORR) after treatment was 82.8% (90% CI, 67.1‐93.0%). The lower boundary of 90% CI for response rates was above the threshold of 35%, thus meeting the protocol‐specified criterion of response rate to dose‐adjusted GDP‐R (*P* < .001). The CR rate was 58.6% (90% CI, 41.7‐74.1%). Regarding disease status at study entry, the ORR was 88.8% with relapse after more than 1 year after initial therapy (CR was achieved by 66.6%), 75.0% with relapse within less than 1 year after initial therapy (CR was achieved by 50.0%), and 66.6% with primary induction failure (CR was achieved by 33.3%). At a median follow‐up of 20.9 months (range: 1.2‐41.3 months), the 2‐year PFS and OS rates were 46.8% (90% CI, 30.7‐61.5%; Figure 1) and 63.2% (90% CI, 45.8‐76.3%; Figure 2), respectively. The OS associated with the interim response after three cycles of treatment (CR + PR vs SD + PD) was significantly different (*P* < .001; Figure S1). In univariate analysis using prognostic variables, none of the following variables was predictive of PFS or OS to treatment: age, sex, IPI, and pathological markers, including non‐GCB, CD5, MIB‐1, and Bcl2 and c‐Myc IHC/FISH status. Only p53 status was predictive of both PFS and OS (Table 2).

**TABLE 2:** Univariable analysis of factors for PFS and OS in DLBCL patients

	PFS			OS		
Variable	HR	95% CI	*P*‐value	HR	95% CI	P‐value
Age > 75 years	0.653	0.263‐1.620	.358	0.692	0.232‐2.066	.510
Male	0.944	0.378‐2.357	.902	1.464	0.470‐4.561	.511
IPI HI and H‐risk	2.701	1.000‐7.297	.051	2.796	0.825‐9.471	.099
Non‐GCB	1.670	0.545‐5.119	.370	1.399	0.381‐5.143	.613
P53‐IHC > 20%	3.954	1.358‐11.511	.012	4.042	1.164‐14.036	.028

*Note*. Univariable Cox proportional hazards regression analysis was applied for PFS and OS.

Abbreviations: CI, confidence interval; DLBCL, diffuse large B‐cell lymphoma; GCB, germinal center B‐cell‐like; H, high; HI, high‐intermediate; HR, hazard ratio; IHC, immunohistochemistry; IPI, international prognostic index; OS, overall survival; PFS, progression‐free survival.

### 3.3 Toxicity assessments, dose adjustment, and QOL

**FIGURE 3:**
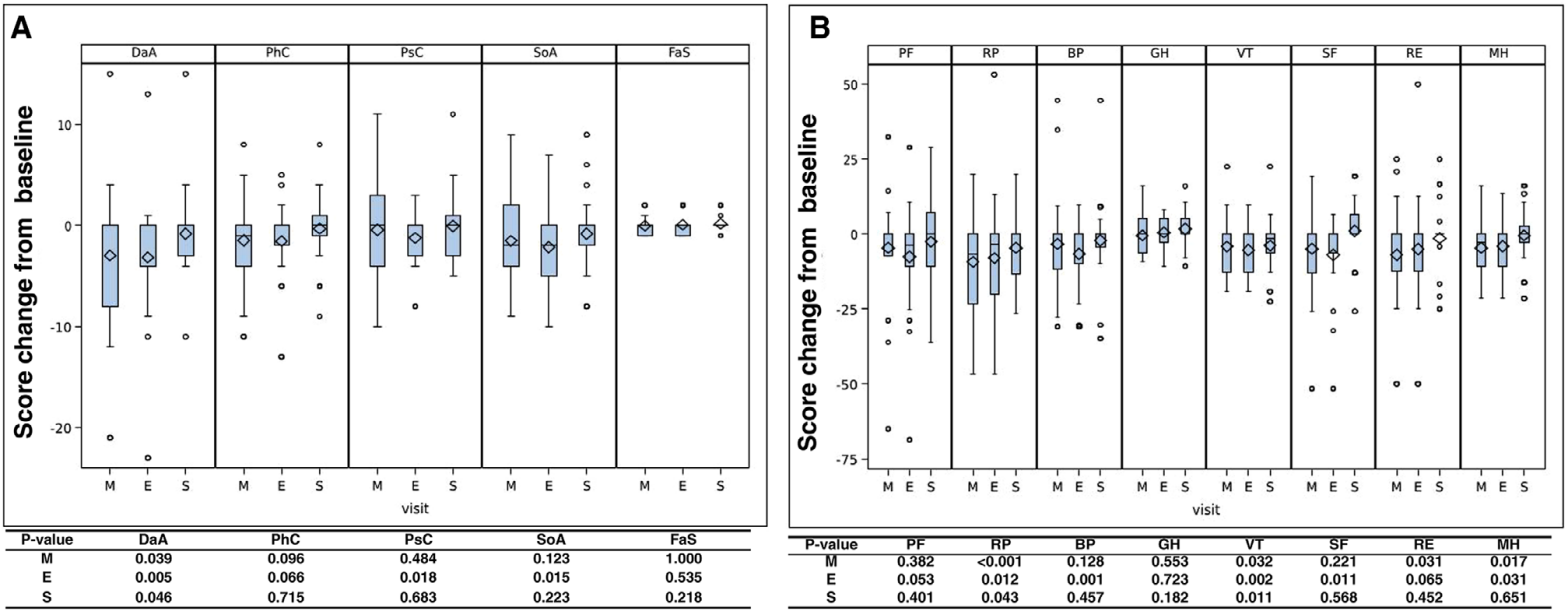
Box and whisker plot of total quality of life (QOL) scores using QOL‐ACD (A) and SF‐36 health survey (B). The bottom and top of the box are the 25th and 75th percentiles, respectively. The thick band and the square near the middle of the box are the 50th percentile (the median) and the mean, respectively. The ends of the whiskers represent the lowest datum still within 1.5 of the interquartile range (IQR) of the lower quartile, and the highest datum still within 1.5 of the IQR of the upper quartile. The open circles are outliers between 1.5 and 3 of the IQR from the end of a box. QOL was assessed at baseline, middle (M), end of the protocol (E), and 6 months after the end of the protocol (S). A statistically significant change (*P*‐value) in a QOL score compared with the baseline score is shown under the figures. BP, bodily pain; DaA, daily activity; FaS, face scale; GH, general health perception; MH, general mental health; N, number of patients; PF, physical functioning; PhC, physical condition; PsC, psychological condition; RE, role of limitations caused by personal or emotional health problems; RP, role limitations caused by physical health problems; SF, social functioning; SoA, social attitude; VT, vitality

Thirty‐three patients constituted the safety analysis set. All AEs including grade 3 and 4 are summarized in Table 3. Sixteen severe AEs including common grade 4 white blood cell decrease (n = 1), grade 4 neutrophil count decrease (n = 2), grade 3 febrile neutropenia (n = 2), grade 4 lung infection (n = 1), grade 1 (n = 1) and grade 2 (n = 1) skin and subcutaneous tissue disorders, grade 1 diarrhea (n = 1), grade 3 lower gastrointestinal hemorrhage (n = 1), grade 3 venous thrombosis (n = 1), grade 3 (n = 1) and grade 4 (n = 1) hyponatremia, grade 3 hyperglycemia (n = 2), and grade 2 creatinine increase (n = 1) were observed in 10 patients, which improved with supportive treatment. Almost all severe AEs occurred in the early cycles (cycles 1‐2 of GDP‐R). Common AEs were hematological toxicities and febrile neutropenia. No patient died because of protocol treatment‐related complications. Three patients discontinued treatment after one cycle because of AEs: pneumonia, hyponatremia, or elevation of creatinine. Dose reductions in each cycle of treatment are shown in Figure S2. The dose of gemcitabine, dexamethasone, and cisplatin was reduced in three of 33 patients (9%), and the dose of gemcitabine and cisplatin was reduced in four of 33 patients (12%). Regarding QOL assessment (n = 29) using QOL‐ACD and SF‐36 health surveys, changes from baseline status are shown in Figures 3A and 3B. Using the QOL‐ACD, statistically significant differences were found in daily activity, psychological condition, and social attitude scores of patients, comparing the middle and end of therapy. Except for daily activity, other scores in the QOL‐ACD were significantly improved from baseline to final scores. Regarding the SF‐36 health survey, there were statistically significant changes in body pain and vitality from baseline to final scores.

**TABLE 3:** Adverse events (n = 33)

	Number of patients (%)		
Toxicity	Any grade	Grade 3	Grade 4
Hematological toxicities			
WBC decreased	28 (84.8)	11 (33.3)	15 (45.5)
Neutrophil count decreased	28 (84.8)	5 (15.2)	21 (63.6)
Lymphocyte count decreased	25 (75.8)	9 (27.3)	13 (39.4)
Anemia	28 (84.8)	16 (48.5)	1 (3.0)
Platelet count decreased	28 (84.8)	4 (12.1)	19 (57.6)
Infections			
Febrile neutropenia	13 (39.4)	13 (39.4)	0
Lung infection	2 (6.1)	1 (3.0)	1 (3.0)
Pruritus	2 (6.1)	0	0
Nonhematological toxicities			
Constitutional symptoms			
Fatigue	6 (18.2)	3 (9.1)	0
Malaise	24 (72.7)	0	0
Fever	11 (33.3)	0	0
Weight gain	10 (30.3)	0	0
Weight loss	11 (33.3)	0	0
Dermatological			
Skin and subcutaneous tissue disorders	2 (6.1)	0	0
Gastrointestinal			
Anorexia	20 (60.6)	2 (6.1)	0
Nausea	15 (45.5)	1 (3.0)	0
Vomiting	5 (15.2)	0	0
Constipation	19 (57.6)	0	0
Diarrhea	7 (21.3)	0	0
Mucositis oral	1 (3.0)	0	0
Ileus	1 (3.0)	0	0
Lower gastrointestinal hemorrhage	1 (3.0)	1 (3.0)	0
Neurological			
Peripheral motor neuropathy	1 (3.0)	0	0
Peripheral sensory neuropathy	6 (18.2)	0	0
Cardiac/vascular			
Hypotension	4 (12.1)	1 (3.0)	0
Venous thrombosis	1 (3.0)	1 (3.0)	0
Urinary			
Hematuria	2 (6.1)	1 (3.0)	0
Laboratory values/chemistries			
Hyponatremia	2 (6.1)	1 (3.0)	1 (3.0)
Hyperglycemia	9 (27.3)	4 (12.1)	0
Hypoalbuminemia	16 (48.5)	2 (6.1)	0
AST increased	15 (45.5)	2 (6.1)	0
ALT increased	13 (39.4)	1 (3.0)	0
Blood bilirubin increased	1 (3.0)	0	0
Creatinine increased	12 (46.4)	0	0

Abbreviations: ALT, alanine aminotransferase; AST, aspartate aminotransferase; WBC, white blood cell.

### 3.4 Cost and ratio of inpatient hospital admissions and outpatient visits

The mean cumulative total direct medical costs were 4 623 980 Japanese yen (range: 964 320‐9 806 250 yen). The total median period of treatment was 177 days (range: 17‐331 days). The median period of patient admission to hospital was 70 days (range: 14‐102 days). The median period of outpatient visits for treatment was 81 days (range: 0‐218 days). The median ratio of inpatient hospital admissions and outpatient visits was 60% (range: 10‐80%).

## 4 DISCUSSION

To the best of our knowledge, this is the first study to evaluate dose‐adjusted GDP‐R for ASCT‐ineligible elderly patients with R/R DLBCL. The results confirmed our hypothesis that dose‐adjusted GDP‐R is an effective and feasible salvage regimen for elderly patients. The primary endpoint of the trial was met, and the ORR of dose‐adjusted GDP‐R was more effective than that found in previous trials.

The population in this study was highly pretreated, including R‐CHOP, and patients had high‐risk DLBCL with a poor long‐term prognosis. The ORR was 82.8%, which is high, considering the R‐CHOP pretreatment, the high‐risk characteristics, and the poor prognosis of the study population. In our study, 58.1% of patients experienced recurrence after 1 year of completing primary therapy. Regarding the cell of origin, 80.6% of patients had the GCB subtype of DLBCL. Regarding pathological markers, we observed one patient who was double positive for Bcl‐2‐FISH and c‐Myc‐FISH. Selection bias may appear to account for the favorable results achieved with dose‐adjusted GDP‐R in the current study. In the trial by Crump et al [7], 27% of patients had relapsed after 1 year and approximately 30% had primary refractory disease. Additionally, in our study, p53 status was predictive of PFS and OS. Mutations in p53, a cause of drug expression in vitro, were assessed using p53 overexpression as a surrogate, which was significantly associated with decreased survival [17]. Several second‐line chemotherapy regimens designed to increase the proportion of ASCT‐ineligible elderly patients with R/R DLBCL through the use of more intense chemotherapy were associated with substantial hematologic toxicity and considerable use of healthcare resources [18]. In our study, p53 expression levels correlated with PFS and OS, and highlighted the potential clinical effectiveness of using bendamustine in combination with rituximab (BR) [19], which will be evaluated in the treatment of lymphoma with higher p53 expression levels in the near future. Recently, polatuzumab vedotin combined with BR resulted in a significantly higher CR rate and reduced the risk of death by 58% compared with BR in patients with transplantation‐ineligible R/R transplant‐ineligible DLBCL [20], but this has not been confirmed.

Prognosis of patients with R/R DLBCL is generally poor; drugs for salvage therapy have some cross‐resistance to standard chemotherapy regimens such as R‐CHOP. Regimens that contain an anthracycline such as doxorubicin, included in CHOP, are not suitable as second‐line chemotherapy because of the cardiac toxicity arising from the accumulation of anthracycline. The combination of gemcitabine and cisplatin may be an effective salvage therapy for R/R DLBCL after R‐CHOP. However, the optimal dose of GDP‐R has not been determined. The novel use of GDP‐R in this study showed promising response rates in elderly patients with R/R DLBCL. Dose‐adjusted GDP‐R had better outcomes in DLBCL patients because of the prolonged control of the disease after relapse.

The 2‐year PFS rate after treatment with dose‐adjusted GDP‐R was 46.8%, which was noninferior compared with previous trials (Table S1). Hou et al reported that the 2‐year PFS rate in patients with R/R aggressive B‐cell non‐Hodgkin's lymphoma who received R‐GDP was 48.0% [21], but no study has examined the efficacy of R‐GDP therapy in patients aged ≥65 years. A phase II clinical study involving R/R DLBCL patients aged 60‐70 years in Algeria showed that the incidences of grade 3‐4 leukopenia and thrombocytopenia in patients receiving GDP therapy (n = 48) were significantly lower than in those receiving etoposide, cisplatin, cytarabine, and methylprednisolone [8] therapy (n = 48). The 3‐year PFS rates were 20.5% and 10.9%, respectively. Prior to that study, another phase II study of GDP therapy (n = 51) involving patients with R/R DLBCL was conducted in Canada. That study reported a response rate of 49%, and the median PFS was 3.1 months [22]. A large‐scale phase III study compared GDP therapy (n = 310) with DHAP therapy (dexamethasone, cytarabine, and cisplatin: n = 309) as another representative regimen of salvage chemotherapy, and found that the efficacy of GDP therapy was similar to that of DHAP therapy [7]. In the GDP treatment group, the incidences of grade 3‐4 toxicities were lower than in the DHAP treatment group, and the incidence of febrile neutropenia, the number of patients requiring platelet transfusion, and the number of episodes requiring hospital admission were lower. Dose‐adjusted GDP‐R seems to be feasible because grade 3‐4 leukopenia, thrombocytopenia, and febrile neutropenia were tolerable, and no treatment‐related deaths were observed in our study.

Our study had several limitations. First, it was a nonrandomized phase II study with a small number of patients, which was not registered with a planned sample size. Second, 90.3% of the patients had a good PS (<2). Third, considering the feasibility of the clinical trial, this study was not designed to assess PFS as a primary endpoint. Nevertheless, our results showed promising efficacy and manageable toxicity of dose‐adjusted GDP‐R for ASCT‐ineligible elderly patients with R/R DLBCL, and this trial is an important initial step in developing a tailor‐made treatment strategy for elderly patients. To assess the QOL in elderly patients, we observed patient‐oriented QOL, duration of hospitalization, and total medical cost associated with dose‐adjusted GDP‐R treatment, which has clinically meaningful efficacy in elderly patients with R/R DLBCL. Comparing the results of the present study with published data is difficult, because no studies have been published to date in which dose‐adjusted GDP‐R was administered to elderly patients with R/R DLBCL, which was well tolerated without unexpected AEs or treatment‐related deaths.

In summary, this novel therapy was well tolerated; incidence, severity, and type of AEs were acceptable compared with those observed previously [8, 19]. Dose‐adjusted GDP‐R can be considered the preferred treatment option for ASCT‐ineligible elderly patients with R/R DLBCL; however, this issue warrants confirmation in a larger number of patients.

## AUTHOR CONTRIBUTIONS

SY contributed to the study design, data analysis, and manuscript preparation. AK, IC, HI, NS, NH, MS, TS, TK, TY, IY, SY, KS, TH, HT, KO, MT, AS, HI, and HN reviewed the manuscript. AS was responsible for data management. AK was responsible for statistical analysis.

## ETHICS APPROVAL AND CONSENT TO PARTICIPATE

The protocol of this study was approved by the Clinical Research Central Ethics Review Board of the National Hospital Organization in October 2014. This study has been registered in the Clinical Trial Registry (UMIN‐CTR) (UMIN000015492). Prior to this study, the principal investigator or investigators obtained written informed consent based on patient free will.

## CONFLICT OF INTEREST

KO is a consultant to SRL. HI received research funding from Chugai. NS received research funding from Ono, A2 Healthcare, Astellas, Janssen, Merck Sharp & Dohme, Otsuka, Pfizer, PPD SNBL, Sumitomo Dainippon Pharma, Daiichi Sankyo, and Bristol‐Myers Squibb. IY and SY received research funding from Kyowa Kirin and Chugai. KS received research funding from Novartis, GlaxoSmithKline, Janssen, Abbvie, Takeda, Sanofi, Bristol‐Myers Squibb, Ono, Merck Sharp & Dohme, Alexion, Daiichi Sankyo, and Celgene. OK received research funding from Kyowa Kirin, Chugai, Celgene, and Daiichi Sankyo. HN received research funding from Bayer Yakuhin, Takeda, Kyowa‐Kirin, Esai, Bristol‐Myers Squibb, Ono, Zenyaku Kogyo, Solasia, AstraZeneca, SymBio, IQVIA service Japan, Mundipharma, Chugai, and Janssen. HI received honoraria from Novartis, Celgne, Astellas, and Janssen. IY received honoraria from Celgene, Mundipharma, Bristol‐Myers Squibb, Shire Japan, Jansen, Mochida, Merck Sharp & Dohme, Takeda, and Taiho and grants and personal fees from Kyowa Kirin and Chugai. SY received honoraria from Bristol‐Meyers Squibb, Takeda, Eisai, Otsuka, and Celgene. KS received honoraria from Takeda, Bristol‐Myers Squibb, Ono, and Celgene. HN received honoraria from Ono, Celgene, Takeda, Esai, Bristol‐Myers Squibb, Chugai, Mundipharma, Sanofi, Janssen, Zenyaku Kogyo, Novartis, Merck Sharp & Dohme, and Chordia Therapeutics. AK received personal fees from Bayer Yakuhin as a member of the data monitoring committee. All other authors declare no conflict of interest.

## Supporting information

Supporting Information

## Data Availability

De‐identified data are available upon request.
