# Supplementary figures and images for: Phase II study of dose‐adjusted gemcitabine, dexamethasone, cisplatin, and rituximab in elderly relapsed diffuse large B‐cell lymphoma patients
Source: EJHaem. 2020 Oct 15;1(2):507–16. doi: 10.1002/jha2.111 (PMC9176064; doi:10.1002/jha2.111)

## Slide 1
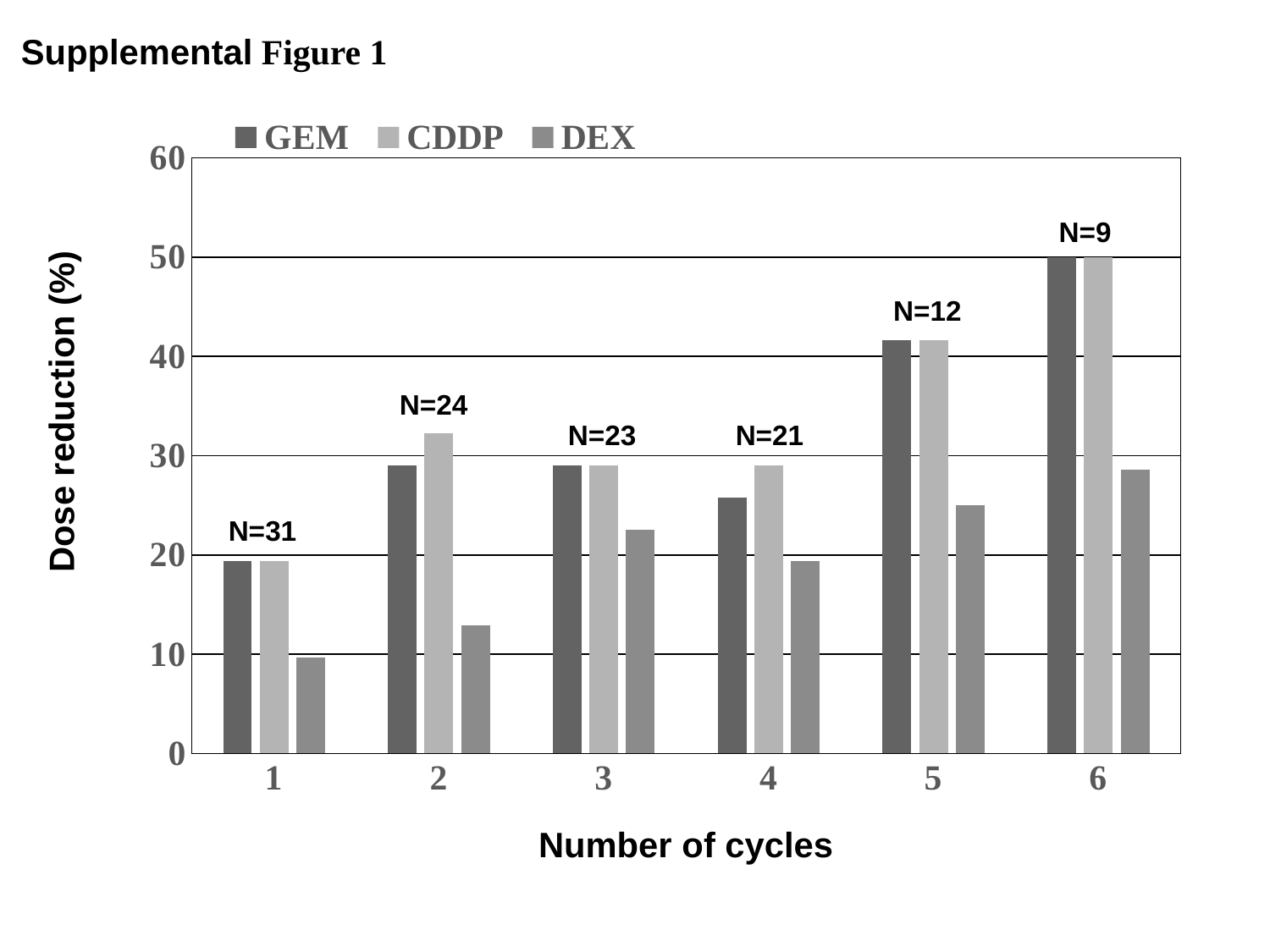

Supplemental Figure 1
### Chart
| Category | GEM | CDDP | DEX |
|---|---|---|---|
| 1 | 19.35 | 19.35 | 9.68 |
| 2 | 29.03 | 32.26 | 12.9 |
| 3 | 29.03 | 29.03 | 22.58 |
| 4 | 25.81 | 29.03 | 19.35 |
| 5 | 41.66 | 41.66 | 25.0 |
| 6 | 50.0 | 50.0 | 28.57 |N=9
N=12
N=24
Dose reduction (%)
N=23
N=21
N=31
Number of cycles

Supplement: Supplementary file 1 — Supporting Information [file JHA2-1-507-s001.pptx]
